# An inflammation-nutrition-surgery-based INSEP model and a web-based clinical decision support system for predicting early postoperative infection in elderly patients with hepatocellular carcinoma: a multicenter retrospective study

**DOI:** 10.3389/fnut.2026.1883480

**Published:** 2026-09-22

**Authors:** Guang-Zhao Shao, Dian-Dian Hao, Fei-Xiang Luo, De-Sheng Zhang, Quan-Zhi Wu, Guo-Tao Fan, Hao-Tian Zheng, Shi-Fei Song, Wei Qiu, Yu-Guo Chen

**Affiliations:** 1 Department of Hepatobiliary and Pancreatic Surgery, General Surgery Center, The First Hospital of Jilin University, Changchun, China; 2 Center for Infectious Diseases and Pathogenic Biology/Department of Hepatology, The First Hospital of Jilin University, Changchun, China; 3 Department of Oncology, Huaihe Hospital of Henan University, Kaifeng, China; 4 Department of General Surgery, Yanshi People's Hospital, Luoyang, Henan, China; 5 Department of General Surgery, The First Affiliated Hospital of Zhengzhou University, Zhengzhou, Henan, China

**Keywords:** early postoperative infection, elderly hepatocellular carcinoma, INSEP model, nomogram, nutritional status, risk prediction, systemic inflammation

## Abstract

**Background:**

Early postoperative infection (EPI) is common in elderly patients with hepatocellular carcinoma (HCC) and is associated with poor prognosis. Effective risk prediction tools are urgently needed for early identification. This study aimed to develop and validate an inflammation-, nutrition-, and surgery-based INSEP nomogram model for predicting EPI-related adverse events in this population.

**Methods:**

A total of 988 elderly HCC patients from two medical centers were retrospectively enrolled and randomly divided into a training dataset (*n* = 692) and an internal validation dataset (*n* = 296), while 381 patients from a third medical center served as an external validation dataset. Restricted cubic spline (RCS), receiver operating characteristic (ROC), least absolute shrinkage and selection operator (LASSO), and multivariable logistic regression analyses were performed to identify independent predictors of EPI and develop the INSEP nomogram. An online risk calculator was established, and model performance was assessed using ROC, calibration, decision curve, and clinical impact curve analyses.

**Results:**

The INSEP model incorporated five independent predictors: SII ≥600 (OR 2.73, *p* = 0.023), CONUT ≥ 3 (OR 7.84, *p* < 0.001), ECOG 2–4 (OR 4.96, *p* < 0.001), laparoscopic surgery (OR 0.83, *p* = 0.025), and surgical duration ≥180 min (OR 7.44, *p* < 0.001). The model achieved AUCs of 0.870 in the training dataset, 0.803 in internal validation, and 0.798 in external validation, with good calibration and clinical utility. Two representative case demonstrations confirmed the model's accurate risk stratification: a low-risk patient (predicted EPI 3.3%) had no infection, while a high-risk patient (predicted 97.0%) developed pneumonia. Usability testing by three independent hepatobiliary surgeons (6–10 years of experience) showed efficient operation (25–60 s per case), intuitive interface, and clear output, with no system errors identified.

**Conclusion:**

The INSEP model is a reliable and practical tool for individualized EPI risk assessment in elderly HCC patients undergoing curative hepatectomy, demonstrating good predictive performance, clinical applicability, and user acceptability.

## Introduction

Hepatocellular carcinoma (HCC) is one of the most common malignancies worldwide, with both its incidence and disease burden continuing to increase (1, 2). Curative hepatectomy remains the primary treatment for patients with resectable HCC and offers the best chance for long-term survival (3, 4). However, with the accelerating aging of the global population, an increasing number of elderly patients are undergoing hepatectomy for HCC. Compared with younger patients, elderly individuals often have a higher burden of comorbidities, impaired immune function, and reduced physiological reserve, resulting in diminished postoperative recovery and an increased risk of postoperative complications (3). Among these, early postoperative infection (EPI) is one of the most common and clinically significant adverse events following hepatectomy. It not only prolongs hospital stay and increases healthcare costs but may also adversely affect both short-term recovery and long-term prognosis (5–7). Therefore, accurate preoperative identification of patients at high risk of EPI and implementation of targeted perioperative management strategies are of great clinical importance for improving postoperative outcomes in elderly patients with HCC.

Growing evidence has demonstrated that systemic inflammatory status and nutritional condition play pivotal roles in tumor progression and postoperative recovery (8–10). Several peripheral blood-based inflammatory and nutritional biomarkers, including the systemic immune-inflammation index (SII), controlling nutritional status (CONUT) score, neutrophil-to-lymphocyte ratio (NLR), and prognostic nutritional index (PNI), have been shown to be closely associated with postoperative complications and long-term outcomes in patients with malignant tumors. Notably, in elderly patients, immunosenescence and age-related impairment in metabolic regulation may further amplify the detrimental effects of chronic inflammation and inadequate nutritional reserves on postoperative recovery, making inflammatory and nutritional biomarkers promising tools for predicting adverse postoperative outcomes in this population (11–13).

In addition to patient-related characteristics, perioperative factors also play a critical role in the development of postoperative infections. Surgical approach, operative duration, and intraoperative blood loss may influence the risk of infection by affecting the extent of tissue injury, the systemic stress response, and postoperative immune function. Previous studies have suggested that open surgery is associated with a higher risk of major postoperative complications than minimally invasive procedures (14, 15). However, studies specifically evaluating the risk of EPI in elderly HCC patients remain limited. Most existing studies have focused on a single inflammatory biomarker, nutritional indicator, or perioperative factor, making it difficult to comprehensively characterize the complex clinical profile of elderly patients. Moreover, many currently available prediction models have been developed using single-center datasets with relatively small sample sizes, and some lack rigorous internal validation or independent external validation, limiting their predictive robustness and generalizability across different clinical settings (10, 16, 17). Therefore, there remains an unmet clinical need to develop a comprehensive prediction model that integrates baseline patient characteristics, age-related physiological status, inflammatory response, nutritional status, and surgery-related factors to achieve accurate and clinically applicable risk stratification for elderly HCC patients.

Nomograms have been widely adopted in oncology for individualized risk prediction by integrating multiple clinical variables into an intuitive and practical predictive tool. However, a comprehensive model specifically developed and multicenter-validated for predicting the risk of EPI in elderly HCC patients is still lacking (18–20). Therefore, in the present study, we developed and validated a novel inflammation–nutrition–surgery–based (INSEP) nomogram by integrating inflammatory, nutritional, and surgery-related factors. This model aims to improve the prediction of postoperative EPI in elderly HCC patients through comprehensive risk assessment, thereby facilitating individualized risk stratification, optimizing perioperative management, and providing a theoretical basis for personalized prevention and intervention in high-risk patients.

## Materials and methods

### Study design and patient selection

This multicenter retrospective study collected clinical data from three medical centers in China. The inclusion criteria were as follows: elderly patients (aged ≥60 years, with a consistent definition of “elderly” across all centers) who were pathologically diagnosed HCC between January 2015 and December 2025. Patients with evidence of preoperative infection, hematological disorders, or unexplained fever were excluded.

Initially, 1,347 elderly HCC patients from the First Hospital of Jilin University and the First Affiliated Hospital of Zhengzhou University, as well as 455 patients from Huaihe Hospital of Henan University, were screened for eligibility. After strict application of the exclusion criteria, 988 patients from the two model-development centers were finally included for model construction and internal validation. Meanwhile, 381 patients from Huaihe Hospital of Henan University were enrolled as an independent external validation dataset to evaluate the generalizability of the model.

This study was approved by the Ethics Committee of the First Hospital of Jilin University and was conducted in accordance with the principles of the Declaration of Helsinki. All data were collected and processed under strict privacy protection measures. Given the retrospective design of this study, all patient data were anonymized, and no personally identifiable information was disclosed; therefore, the requirement for informed consent was waived. Patients were excluded if they met any of the following criteria: (1) receiving preoperative HCC-related treatment; (2) recurrent HCC; (3) preoperative tumor rupture; or (4) missing key variables required for analysis, including the systemic immune-inflammation index (SII), controlling nutritional status (CONUT) score, operative time, or intraoperative blood loss.

Finally, 988 patients were included for model development and internal validation. These patients were randomly assigned to the training dataset (*n* = 692, 70%) and the internal validation dataset (*n* = 296, 30%). A total of 381 patients from Huaihe Hospital of Henan University constituted the independent external validation dataset (Figure 1).

**Figure 1:**
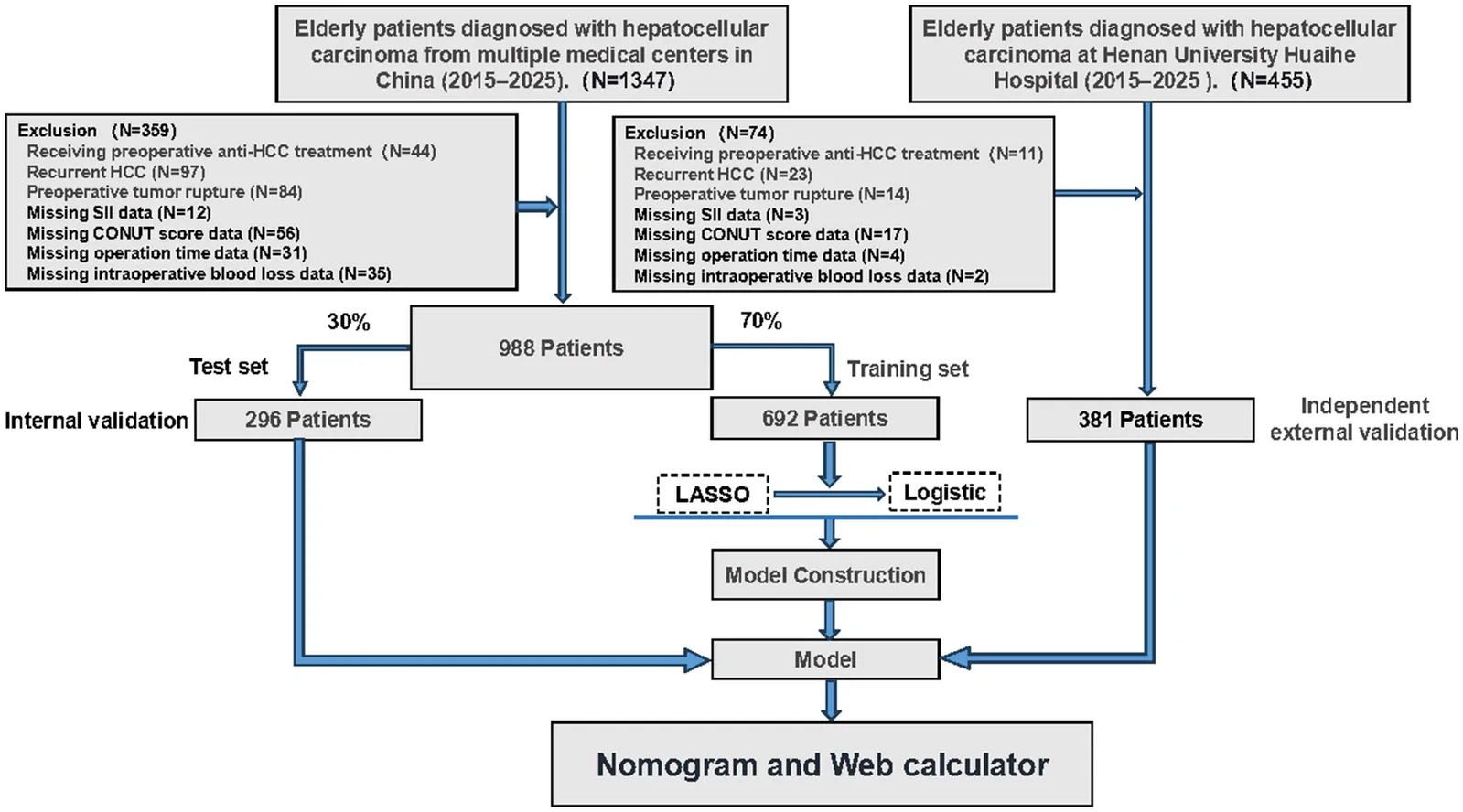
Flowchart showing patients screening process.

To ensure consistency in variable definitions, all candidate predictors and outcome measures were standardized across different study centers. The clinical data were extracted from electronic medical records by trained researchers and integrated into a comprehensive database for statistical analysis. Only patients with complete data for all study variables were included to ensure data integrity and the reliability of the predictive model.

### Data collection and variable definitions

The variables collected in this study included demographic information [sex, age, and body mass index (BMI)], performance status and medical history [Eastern Cooperative Oncology Group (ECOG) performance status, smoking history, alcohol consumption, diabetes, liver disease history, and cirrhosis], and liver function and tumor-related parameters [Child-Pugh grade, Barcelona Clinic Liver Cancer (BCLC) stage, and preoperative alpha-fetoprotein (AFP)]. Preoperative laboratory parameters, including platelet count, neutrophil count, lymphocyte count, albumin, and total cholesterol, were also recorded. Tumor- and surgery-related variables included tumor location, maximum tumor diameter, tumor number, surgical approach, surgical duration, intraoperative blood loss, and intraoperative transfusion.

Composite inflammation- and nutrition-related indices were calculated as follows: the SII index was defined as platelet count × neutrophil count/lymphocyte count, and the CONUT score was calculated as the sum of albumin score, lymphocyte score, and cholesterol score (20).

In this study, operative duration was defined as the actual intraoperative time, referring to the period from the initiation of the surgical procedure to its completion. Receiver operating characteristic (ROC) curve analysis was performed to evaluate the predictive performance of the SII and operative duration for EPI, and the optimal cutoff values were determined using the Youden index. Furthermore, to minimize potential confounding bias associated with the surgical learning curve, early surgical cases performed by surgeons who had not yet acquired sufficient independent operative experience were excluded from the analysis (18).

The primary outcome of this study was EPI, defined as any newly developed infectious event occurring within 7 days after surgery. Infectious events included those diagnosed during postoperative hospitalization and those identified during follow-up within 7 days after surgery that met the predefined diagnostic criteria.

All postoperative infectious events were adjudicated according to a prespecified standardized diagnostic protocol, which was consistently applied across all participating centers. The diagnostic criteria for infections were based on internationally recognized surveillance systems, particularly the definitions and principles established by the Centers for Disease Control and Prevention (CDC)/National Healthcare Safety Network (NHSN). The diagnosis of infection was determined based on a comprehensive assessment of clinical manifestations, microbiological findings, laboratory parameters, and imaging examinations (21, 22).

The types of infections included, but were not limited to, surgical site infection (SSI), pulmonary infection, intra-abdominal infection, urinary tract infection, and bloodstream infection. Detailed diagnostic criteria and adjudication procedures for each type of infection are provided in Supplementary Table 1 to improve methodological transparency and reproducibility. All suspected infection cases were independently reviewed by two experienced physicians according to the predefined diagnostic criteria. In cases of disagreement between the two reviewers, a third senior clinician independently reassessed the case and made the final decision to ensure consistency and accuracy in infection classification.

Other clinical outcomes included length of hospital stay, postoperative intensive care unit (ICU) admission, occurrence of postoperative complications, and complication severity. The severity of postoperative complications was assessed according to the Clavien–Dindo classification system, with grade I–II complications defined as minor complications and grade ≥III complications defined as major complications (23).

### Statistical analysis

All statistical analyses were performed using IBM SPSS Statistics and R software (version 4.6.1). Continuous variables were expressed as mean ± standard deviation (M ± SD) according to their distribution characteristics, and comparisons between groups were performed using the independent-samples *t*-test. Categorical variables were presented as frequencies and percentages and compared using the chi-square test (χ^2^ test). Initially, a correlation heatmap was generated to visualize the pairwise correlations among candidate predictors and to explore potential correlation patterns. In addition, the variance inflation factor (VIF) was calculated to quantitatively assess multicollinearity among candidate variables. Generally, higher VIF values indicate a greater possibility of problematic multicollinearity.

To further investigate potential dose–response relationships between continuous variables and the risk of EPI, RCS regression models were applied to evaluate the associations of the SII index and operative duration with EPI risk. Nonlinearity tests were performed to determine whether significant nonlinear associations existed between continuous predictors and outcomes. Subsequently, receiver operating characteristic (ROC) curve analysis was conducted to assess the predictive performance of SII and operative duration for EPI, and the area under the curve (AUC) was calculated. The optimal cutoff values were determined using the Youden index, and continuous variables were dichotomized according to these cutoff values for subsequent model development. To reduce redundant variables and identify clinically relevant predictors associated with EPI, least absolute shrinkage and selection operator (LASSO) regression was performed. All candidate variables were entered into the LASSO model. Ten-fold cross-validation was applied to determine the optimal penalty parameter (λ), and variables selected based on the cross-validation results were retained as candidate predictors.

The variables selected by LASSO regression were subsequently incorporated into multivariable logistic regression analysis to identify independent predictors of EPI and develop the INSEP prediction model. Considering the multicenter design of this study, potential heterogeneity among participating centers, including differences in baseline characteristics, perioperative management strategies, and surgical procedures, was considered. Therefore, study center was included as a categorical covariate in the multivariable logistic regression model to adjust for potential center effects.

Based on the independent predictors identified by multivariable logistic regression, an individualized risk prediction nomogram was developed using the “rms” package in R software to estimate the probability of EPI occurrence for individual patients. In addition, an online risk calculator based on the Shiny framework was developed to facilitate clinical implementation of the model. Model performance was evaluated in terms of discrimination, calibration, and clinical utility. Discrimination was assessed using ROC curves and AUC values. Calibration was evaluated using calibration plots, calibration intercepts, calibration slopes, and Brier scores to assess the agreement between predicted probabilities and observed outcomes. Decision curve analysis (DCA) and clinical impact curves (CIC) were performed to evaluate the clinical net benefit and potential applicability of the model. The DeLong test was used to compare differences in AUC values between the INSEP model and individual predictors to assess the incremental predictive value of the combined model. All statistical tests were two-sided, and a *P* value < 0.05 was considered statistically significant.

## Results

### Clinical characteristics of patients

According to the predefined inclusion and exclusion criteria, a total of 1,369 elderly HCC patients were included in this study. Among them, 988 patients from the First Hospital of Jilin University and the First Affiliated Hospital of Zhengzhou University were randomly assigned to the training dataset (*n* = 692) and the internal validation dataset (*n* = 296) at a ratio of 7:3 (Figure 1). In the training dataset, RCS analysis was performed to further explore the potential dose–response relationships between the SII, operative duration, and the risk of EPI. No significant nonlinear associations were observed between SII or operative duration and EPI risk, with *P* values for nonlinearity of 0.233 and 0.905, respectively (Supplementary Figures 1A, B).

Subsequently, ROC curve analysis was conducted to evaluate the predictive performance of SII and operative duration for EPI. Both variables demonstrated potential predictive value. The optimal cutoff values determined by ROC analysis were 597 for SII and 178 min for operative duration (Supplementary Figures 2A, B). Considering that the SII cutoff of 597 was close to 600, and that rounding to 600 had minimal impact on model discrimination while improving clinical applicability, the final cutoff value for SII was set at 600. Similarly, the cutoff value for operative duration was rounded to 180 min. These predefined cutoff values were subsequently used for EPI stratification and univariable and multivariable logistic regression analyses. As shown in Table 1, baseline characteristics were generally well balanced between the training and internal validation datasets, with no statistically significant differences observed among all variables (all *P* > 0.05).

**Table 1:** Baseline characteristics of elderly patients with hepatocellular carcinoma in the training and internal validation datasets.

Variables	Categories	Total (*N* = 988)	Training dataset (*N* = 692)	Internal validation dataset (*N* = 296)	*P* value
Demographic data					
Gender (%)	Male	862 (87.25)	599 (86.56)	263 (88.85)	0.376
	Female	126 (12.75)	93 (13.44)	33 (11.15)	
Age (%)	< 70	445 (45.04)	319 (46.10)	126 (42.57)	0.341
	≥70	543 (54.96)	373 (53.90)	170 (57.43)	
BMI (%)	Under weight	104 (10.53)	74 (10.69)	30 (10.14)	0.564
	Normal weight	532 (53.85)	365 (52.75)	167 (56.42)	
	Overweight/Obesity	352 (35.63)	253 (36.56)	99 (33.45)	
ECOG (%)	0–1	720 (72.87)	509 (73.55)	211 (71.28)	0.511
	2–4	268 (27.13)	183 (26.45)	85 (28.72)	
Smoke (%)	No	611 (61.84)	419 (60.55)	192 (64.86)	0.227
	Yes	377 (38.16)	273 (39.45)	104 (35.14)	
Drink (%)	No	732 (74.09)	509 (73.55)	223 (75.34)	0.612
	Yes	256 (25.91)	183 (26.45)	73 (24.66)	
DM (%)	No	881 (89.17)	616 (89.02)	265 (89.53)	0.901
	Yes	107 (10.83)	76 (10.98)	31 (10.47)	
History of liver disease (%)	No	905 (91.60)	641 (92.63)	264 (89.19)	0.097
	Yes	83 (8.40)	51 (7.37)	32 (10.81)	
Cirrhosis (%)	No	271 (27.43)	195 (28.18)	76 (25.68)	0.465
	Yes	717 (72.57)	497 (71.82)	220 (74.32)	
Child pugh grade (%)	No	902 (91.30)	629 (90.90)	273 (92.23)	0.579
	Yes	86 (8.70)	63 (9.10)	23 (7.77)	
BCLC stage (%)	0	598 (60.53)	428 (61.85)	170 (57.43)	0.386
	A	167 (16.90)	111 (16.04)	56 (18.92)	
	B	223 (22.57)	153 (22.11)	70 (23.65)	
Laboratory data					
SII (%)	< 600	870 (88.06)	610 (88.15)	260 (87.84)	0.975
	≥600	118 (11.94)	82 (11.85)	36 (12.16)	
CONUT (%)	< 3	773 (78.24)	543 (78.47)	230 (77.70)	0.855
	≥3	215 (21.76)	149 (21.53)	66 (22.30)	
Preoperative AFP (%)	< 400	713 (72.17)	511 (73.84)	202 (68.24)	0.085
	≥400	275 (27.83)	181 (26.16)	94 (31.76)	
Imaging data					
Tumor location (%)	Right lobe	289 (29.25)	207 (29.91)	82 (27.70)	0.543
	Left lobe	575 (58.20)	395 (57.08)	180 (60.81)	
	Bilateral	124 (12.55)	90 (13.01)	34 (11.49)	
Maximum tumor diameter (%)	< 5	289 (29.25)	207 (29.91)	82 (27.70)	0.5425
	5–10	575 (58.20)	395 (57.08)	180 (60.81)	
	≥10	124 (12.55)	90 (13.01)	34 (11.49)	
Number of tumors (%)	Single	834 (84.41)	587 (84.83)	247 (83.45)	0.651
	Multiple	154 (15.59)	105 (15.17)	49 (16.55)	
Perioperative information					
Operative approach (%)	Open	575 (58.20)	385 (55.64)	190 (64.19)	0.015
	Laparoscopic	413 (41.80)	307 (44.36)	106 (35.81)	
Surgical duration (%)	< 180	703 (71.15)	483 (69.80)	220 (74.32)	0.173
	≥180	285 (28.85)	209 (30.20)	76 (25.68)	
Intraoperative blood loss (%)	< 400	632 (63.97)	440 (63.58)	192 (64.86)	0.755
	≥400	356 (36.03)	252 (36.42)	104 (35.14)	
Intraoperative blood transfusion (%)	No	799 (80.87)	558 (80.64)	241 (81.42)	0.842
	Yes	189 (19.13)	134 (19.36)	55 (18.58)	
Length of hospital stay [mean (SD)]		11.523 (7.56)	11.412 (6.72)	11.784 (9.23)	0.479
ICU admission postoperatively (%)	No	907 (91.80)	631 (91.18)	276 (93.24)	0.340
	Yes	81 (8.20)	61 (8.82)	20 (6.76)	
Minor complications (%)	No	775 (78.44)	544 (78.61)	231 (78.04)	0.907
	Yes	213 (21.56)	148 (21.39)	65 (21.96)	
Major complications (%)	No	893 (90.38)	620 (89.60)	273 (92.23)	0.242
	Yes	95 (9.62)	72 (10.40)	23 (7.77)	
EPI (%)	No	839 (84.92)	580 (83.82)	259 (87.50)	0.165
	Yes	149 (15.08)	112 (16.18)	37 (12.50)	

BMI, body mass index; ECOG, eastern cooperative oncology group; DM, diabetes mellitus; BCLC, barcelona clinic liver cancer; SII, systemic immune-inflammation index; CONUT, controlling nutritional status; AFP, alpha-fetoprotein; ICU, intensive care unit admission; Minor complications, Clavien-Dindo Grade I-II; Major complications, Clavien-Dindo Grade III; EPI, early postoperative infection; CONUT score ≥3 indicates malnutrition.

Baseline characteristics were further compared between the model-development dataset and the independent external validation dataset (*n* = 381; Table 2). Significant differences were observed between the two datasets in several demographic, clinical, and perioperative variables, including sex, age, ECOG performance status, alcohol consumption, diabetes mellitus, cirrhosis, BCLC stage, CONUT score, maximum tumor diameter, surgical approach, operative duration, intraoperative blood loss, intraoperative blood transfusion, length of hospital stay, incidence of minor complications, incidence of major complications, and occurrence of EPI (all *P* < 0.05).

**Table 2:** Comparison of baseline characteristics of elderly patients with hepatocellular carcinoma between the multicenter dataset and the independent validation dataset.

Variables	Categories	Total (*N* = 1,369)	Multi-center dataset (*N* = 988)	Independent validation dataset (*N* = 381)	*P* value
Demographic data					
Gender (%)	Male	1161 (84.81)	862 (87.25)	299 (78.48)	0.001
	Female	208 (15.19)	126 (12.75)	82 (21.52)	
Age (%)	< 70	665 (48.58)	445 (45.04)	220 (57.74)	< 0.001
	≥70	704 (51.42)	543 (54.96)	161 (42.26)	
BMI (%)	Under weight	160 (11.69)	104 (10.53)	56 (14.70)	0.081
	Normal weight	734 (53.62)	532 (53.85)	202 (53.02)	
	Overweight/Obesity	475 (34.70)	352 (35.63)	123 (32.28)	
ECOG (%)	0–1	1037 (75.75)	720 (72.87)	317 (83.20)	0.001
	2–4	332 (24.25)	268 (27.13)	64 (16.80)	
Smoke (%)	No	826 (60.34)	611 (61.84)	215 (56.43)	0.076
	Yes	543 (39.66)	377 (38.16)	166 (43.57)	
Drink (%)	No	978 (71.44)	732 (74.09)	246 (64.57)	0.001
	Yes	391 (28.56)	256 (25.91)	135 (35.43)	
DM (%)	No	1198 (87.51)	881 (89.17)	317 (83.20)	0.003
	Yes	171 (12.49)	107 (10.83)	64 (16.80)	
History of liver disease (%)	No	1255 (91.67)	905 (91.60)	350 (91.86)	0.961
	Yes	114 (8.33)	83 (8.40)	31 (8.14)	
Cirrhosis (%)	No	447 (32.65)	271 (27.43)	176 (46.19)	< 0.001
	Yes	922 (67.35)	717 (72.57)	205 (53.81)	
Child pugh grade (%)	No	1258 (91.89)	902 (91.30)	356 (93.44)	0.234
	Yes	111 (8.11)	86 (8.70)	25 (6.56)	
BCLC stage (%)	0	854 (62.38)	598 (60.53)	256 (67.19)	0.001
	A	198 (14.46)	167 (16.90)	31 (8.14)	
	B	317 (23.16)	223 (22.57)	94 (24.67)	
Laboratory data					
SII (%)	< 600	1195 (87.29)	870 (88.06)	325 (85.30)	0.200
	≥600	174 (12.71)	118 (11.94)	56 (14.70)	
CONUT (%)	< 3	1097 (80.13)	773 (78.24)	324 (85.04)	0.006
	≥3	272 (19.87)	215 (21.76)	57 (14.96)	
Preoperative AFP (%)	< 400	993 (72.53)	713 (72.17)	280 (73.49)	0.671
	≥400	376 (27.47)	275 (27.83)	101 (26.51)	
Imaging data					
Tumor location (%)	Right lobe	392 (28.63)	289 (29.25)	103 (27.03)	0.265
	Left lobe	814 (59.46)	575 (58.20)	239 (62.73)	
	Bilateral	163 (11.91)	124 (12.55)	39 (10.24)	
Maximum tumor diameter (%)	< 5	489 (35.72)	365 (36.94)	124 (32.55)	< 0.001
	5–10	639 (46.68)	521 (52.73)	118 (30.97)	
	≥10	241 (17.60)	102 (10.32)	139 (36.48)	
Number of tumors (%)	Single	1149 (83.93)	834 (84.41)	315 (82.68)	0.483
	Multiple	220 (16.07)	154 (15.59)	66 (17.32)	
Perioperative information					
Operative approach (%)	Open	837 (61.14)	575 (58.20)	262 (68.77)	0.001
	Laparoscopic	532 (38.86)	413 (41.80)	119 (31.23)	
Surgical duration (%)	< 180	877 (64.06)	703 (71.15)	174 (45.67)	< 0.001
	≥180	492 (35.94)	285 (28.85)	207 (54.33)	
Intraoperative blood loss (%)	< 400	834 (60.92)	632 (63.97)	202 (53.02)	0.003
	≥400	535 (39.08)	356 (36.03)	179 (46.98)	
Intraoperative blood transfusion (%)	No	1046 (76.41)	799 (80.87)	247 (64.83)	< 0.001
	Yes	323 (23.59)	189 (19.13)	134 (35.17)	
Length of hospital stay [mean (SD)]		12.023 (7.596)	11.523 (7.564)	13.318 (7.534)	0.001
ICU admission postoperatively (%)	No	1261 (92.11)	907 (91.80)	354 (92.91)	0.564
	Yes	108 (7.89)	81 (8.20)	27 (7.09)	
Minor complications (%)	No	949 (69.32)	775 (78.44)	174 (45.67)	< 0.001
	Yes	420 (30.68)	213 (21.56)	207 (54.33)	
Major complications (%)	No	1089 (79.55)	893 (90.38)	196 (51.44)	< 0.001
	Yes	280 (20.45)	95 (9.62)	185 (48.56)	
EPI (%)	No	1187 (86.71)	839 (84.92)	348 (91.34)	0.002
	Yes	182 (13.29)	149 (15.08)	33 (8.66)	

BMI, body mass index; ECOG, eastern cooperative oncology group; DM, diabetes mellitus; BCLC, barcelona clinic liver cancer; SII, systemic immune-inflammation index; CONUT, controlling nutritional status; AFP, alpha-fetoprotein; ICU, intensive care unit admission; minor complications, Clavien-Dindo Grade I-II; major complications, Clavien-Dindo Grade III; EPI, early postoperative infection.

### Comparison of baseline characteristics and clinical outcomes between the EPI and non-EPI groups

As shown in Supplementary Figure 3A, pulmonary infection was the most common type of EPI among elderly HCC patients, accounting for 45% of all infection events, followed by SSI (15%) and intra-abdominal infection (12%). Other types of infection included other infections (11%), urinary tract infection (10%), and sepsis (7%).

Regarding the distribution of causative pathogens, Escherichia coli was the most frequently identified pathogen (33%), followed by Klebsiella pneumoniae (17%) and Pseudomonas aeruginosa (12%). Other detected pathogens included Acinetobacter baumannii (7%), Enterobacter species (4%), and other bacterial species (2%) (Supplementary Figure 3B). According to the occurrence of EPI, patients were further classified into the EPI group (*n* = 182) and the non-EPI group (*n* = 1,187), and baseline characteristics were compared between the two groups (Table 3). No statistically significant differences were observed between the two groups in terms of sex, age, BMI, smoking history, alcohol consumption, diabetes mellitus, history of liver disease, Child–Pugh grade, tumor location, or tumor number (all *P* > 0.05).

**Table 3:** Baseline characteristics between elderly patients with hepatocellular carcinoma with and without EPI.

Variables	Categories	Total (*N* = 1,369)	Non-EPI (*N* = 1187)	EPI (*N* = 182)	*P* value
Demographic data					
Gender (%)	Male	1161 (84.81)	1009 (85.00)	152 (83.52)	0.682
	Female	208 (15.19)	178 (15.00)	30 (16.48)	
Age (%)	< 70	665 (48.58)	574 (48.36)	91 (50.00)	0.739
	≥70	704 (51.42)	613 (51.64)	91 (50.00)	
BMI (%)	Under weight	160 (11.69)	137 (11.54)	23 (12.64)	0.176
	Normal weight	734 (53.62)	627 (52.82)	107 (58.79)	
	Overweight/Obesity	475 (34.70)	423 (35.64)	52 (28.57)	
ECOG (%)	0–1	1037 (75.75)	928 (78.18)	109 (59.89)	< 0.001
	2–4	332 (24.25)	259 (21.82)	73 (40.11)	
Smoke (%)	No	826 (60.34)	712 (59.98)	114 (62.64)	0.548
	Yes	543 (39.66)	475 (40.02)	68 (37.36)	
Drink (%)	No	978 (71.44)	840 (70.77)	138 (75.82)	0.187
	Yes	391 (28.56)	347 (29.23)	44 (24.18)	
DM (%)	No	1198 (87.51)	1044 (87.95)	154 (84.62)	0.251
	Yes	171 (12.49)	143 (12.05)	28 (15.38)	
History of liver disease (%)	No	1255 (91.67)	1087 (91.58)	168 (92.31)	0.850
	Yes	114 (8.33)	100 (8.42)	14 (7.69)	
Cirrhosis (%)	No	447 (32.65)	402 (33.87)	45 (24.73)	0.018
	Yes	922 (67.35)	785 (66.13)	137 (75.27)	
Child pugh grade (%)	No	1258 (91.89)	1093 (92.08)	165 (90.66)	0.611
	Yes	111 (8.11)	94 (7.92)	17 (9.34)	
BCLC stage (%)	0	854 (62.38)	756 (63.69)	98 (53.85)	0.020
	A	198 (14.46)	170 (14.32)	28 (15.38)	
	B	317 (23.16)	261 (21.99)	56 (30.77)	
Laboratory data					
SII (%)	< 600	1195 (87.29)	1076 (90.65)	119 (65.38)	< 0.001
	≥600	174 (12.71)	111 (9.35)	63 (34.62)	
CONUT (%)	< 3	1097 (80.13)	1017 (85.68)	80 (43.96)	< 0.001
	≥3	272 (19.87)	170 (14.32)	102 (56.04)	
Preoperative AFP (%)	< 400	993 (72.53)	896 (75.48)	97 (53.30)	< 0.001
	≥400	376 (27.47)	291 (24.52)	85 (46.70)	
Imaging data					
Tumor location (%)	Right lobe	392 (28.63)	348 (29.32)	44 (24.18)	0.113
	Left lobe	814 (59.46)	693 (58.38)	121 (66.48)	
	Bilateral	163 (11.91)	146 (12.30)	17 (9.34)	
Maximum tumor diameter (%)	< 5	489 (35.72)	411 (34.63)	78 (42.86)	0.016
	5–10	639 (46.68)	555 (46.76)	84 (46.15)	
	≥10	241 (17.60)	221 (18.62)	20 (10.99)	
Number of tumors (%)	Single	1149 (83.93)	997 (83.99)	152 (83.52)	0.956
	Multiple	220 (16.07)	190 (16.01)	30 (16.48)	
Perioperative information					
Operative approach (%)	Open	837 (61.14)	749 (63.10)	88 (48.35)	0.001
	Laparoscopic	532 (38.86)	438 (36.90)	94 (51.65)	
Surgical duration (%)	< 180	877 (64.06)	791 (66.64)	86 (47.25)	< 0.001
	≥180	492 (35.94)	396 (33.36)	96 (52.75)	
Intraoperative blood loss (%)	< 400	834 (60.92)	741 (62.43)	93 (51.10)	0.005
	≥400	535 (39.08)	446 (37.57)	89 (48.90)	
Intraoperative blood transfusion (%)	No	1046 (76.41)	925 (77.93)	121 (66.48)	0.001
	Yes	323 (23.59)	262 (22.07)	61 (33.52)	
Length of hospital stay [mean (SD)]		12.023 (7.596)	11.107 (5.359)	17.995 (14.370)	< 0.001
ICU admission postoperatively (%)	No	1261 (92.11)	1134 (95.53)	127 (69.78)	< 0.001
	Yes	108 (7.89)	53 (4.47)	55 (30.22)	
Minor complications (%)	No	949 (69.32)	851 (71.69)	98 (53.85)	< 0.001
	Yes	420 (30.68)	336 (28.31)	84 (46.15)	
Major complications (%)	No	1089 (79.55)	997 (83.99)	92 (50.55)	< 0.001
	Yes	280 (20.45)	190 (16.01)	90 (49.45)	

BMI, body mass index; ECOG, eastern cooperative oncology group; DM, diabetes mellitus; BCLC, barcelona clinic liver cancer; SII, systemic immune-inflammation index; CONUT, controlling nutritional status; AFP, alpha-fetoprotein; ICU, intensive care unit admission; minor complications, Clavien-Dindo Grade I-II; major complications, Clavien-Dindo Grade III; EPI, early postoperative infection.

However, significant differences were observed in several clinical characteristics and perioperative variables. Compared with the non-EPI group, patients in the EPI group had poorer ECOG performance status (2–4 points), a higher proportion of cirrhosis, and more advanced BCLC stage (all *P* < 0.05). In addition, the proportions of patients with SII ≥600, CONUT score ≥3, and preoperative AFP ≥400 were significantly higher in the EPI group than in the non-EPI group (all *P* < 0.001). Regarding tumor characteristics, maximum tumor diameter differed significantly between the two groups (*P* = 0.016).

For perioperative factors, patients in the EPI group had a higher proportion of open surgery, longer operative duration (≥180 min), greater intraoperative blood loss (≥400 mL), and a higher rate of intraoperative blood transfusion compared with those in the non-EPI group (all *P* < 0.05). Regarding clinical outcomes (Supplementary Table 2), significant differences were also observed between the EPI and non-EPI groups. Compared with patients without EPI, those with EPI experienced a significantly longer hospital stay (17.99 ± 14.37 days vs. 11.11 ± 5.36 days) and a higher rate of postoperative intensive care unit (ICU) admission (30.22% vs. 4.47%). Furthermore, the incidences of minor complications (46.15% vs. 28.31%) and major complications (49.45% vs. 16.01%) were significantly higher in the EPI group than in the non-EPI group (all *P* < 0.05).

### Identification of risk factors for EPI

To evaluate correlations and potential collinearity among candidate predictors, a heatmap was first generated to visualize pairwise correlations among all candidate variables and explore the underlying correlation patterns (Figure 2). Subsequently, multicollinearity was quantitatively assessed using the variance inflation factor (VIF). All candidate variables showed relatively low VIF values (range, 1.00–1.47), indicating no significant multicollinearity among the predictors (Supplementary Figure 4).

**Figure 2:**
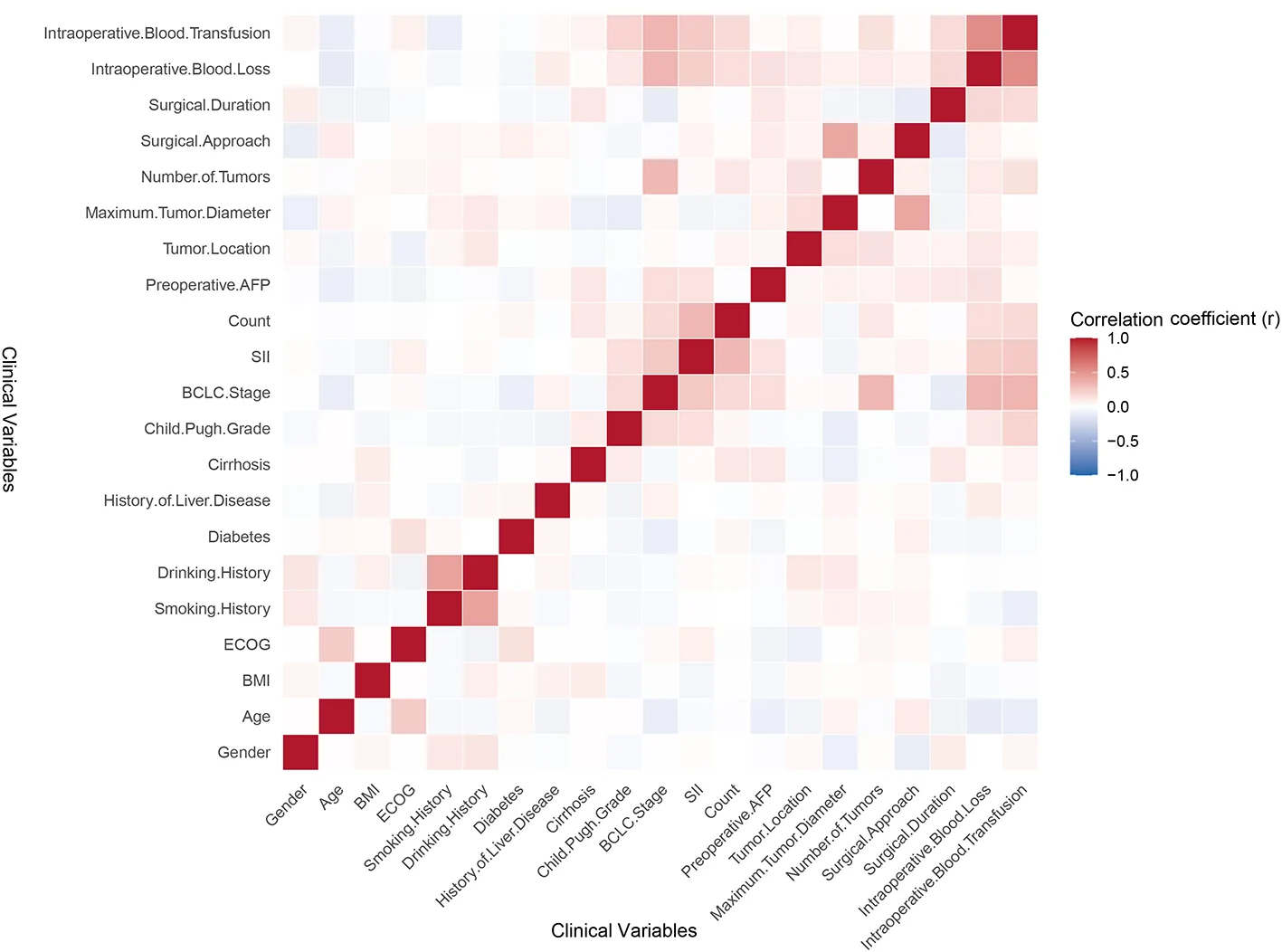
Correlation heatmap of variables.

LASSO regression analysis was subsequently performed to identify important predictors associated with EPI (Figure 3). Candidate variables are presented in Supplementary Table 3. Ten-fold cross-validation was applied to determine the optimal penalty parameter, and the λ value corresponding to the minimum mean cross-validation error (λ.min = 0.00682) was selected as the criterion for variable selection. At this parameter level, LASSO regression identified 13 candidate predictors, including ECOG performance status, drinking history, Child–Pugh grade, BCLC stage, SII, CONUT score, AFP level, tumor location, maximum tumor diameter, number of tumors, operative approach, operative duration, and intraoperative blood loss (Supplementary Table 4). These variables were subsequently entered into multivariable logistic regression analysis to identify independent predictors of EPI and develop the INSEP prediction model.

**Figure 3:**
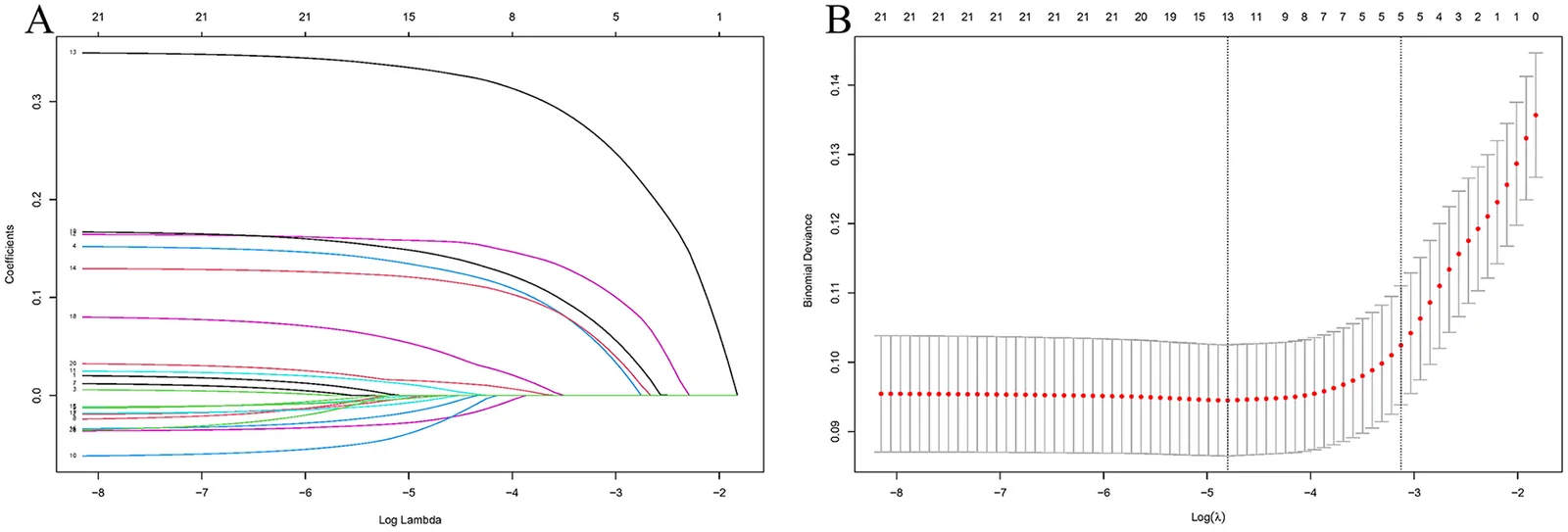
The selection process of the optimum value of the parameter λ in the Lasso regression model by cross-validation method; **(A)** the LASSO path plot; **(B)** the cross-validation plot.

Considering the multicenter design of this study, potential differences in baseline characteristics and perioperative management strategies among participating centers may introduce center effects. Therefore, the impact of center effects on model parameter stability was further evaluated. In the training dataset, study center (the First Hospital of Jilin University and the First Affiliated Hospital of Zhengzhou University) was forced into the multivariable logistic regression model as an adjustment covariate. Compared with the model without center adjustment, changes in the odds ratios (ORs) of all predictors were less than 10%, and the statistical significance of predictors remained largely unchanged (Supplementary Table 5). Moreover, the study center itself was not significantly associated with EPI risk (OR = 1.12, 95% CI: 0.69–1.82, *P* = 0.642). These findings suggested that center effects had no substantial influence on model parameter estimation.

Based on the multivariable logistic regression analysis after adjustment for center effects (Table 4), ECOG performance status, SII, CONUT score, surgical approach, and operative duration were identified as independent risk factors for EPI.

**Table 4:** Risk factors for EPI in elderly HCC patients: multivariable logistic regression analysis adjusted for center effects.

Variables	OR comparison	UV OR (95% CI)	UV *P*	MV OR (95% CI)	MV *P*
Demographic data					
ECOG (%)	0–1	Reference	< 0.001	Reference	< 0.001
	2–4	2.59 (1.70–3.94)		4.96 (2.73–9.00)	
Drink (%)	No	Reference	0.123		
	Yes	1.21 (1.03–3.15)			
Child pugh grade (%)	A	Reference	0.668		
	B	1.85 (1.41–2.78)			
BCLC stage (%)	0	Reference	< 0.001		
	A	1.78 (1.03–3.10)	0.038		
	B	2.39 (1.50–3.81)	< 0.001		
Laboratory data					
SII (%)	< 600	Reference	< 0.001	Reference	0.023
	≥600	7.12 (4.33–11.71)		2.73 (1.43–5.22)	
CONUT (%)	< 3	Reference	< 0.001	Reference	< 0.001
	≥3	8.57 (4.74–14.58)		7.84 (3.46–15.34)	
Preoperative AFP (%)	< 400	Reference	< 0.001		
	≥400	2.89 (1.90–4.41)			
Imaging data					
Tumor location (%)	Right lobe	Reference	0.009		
	Left lobe	1.64 (1.02–2.64)	0.041		
	Bilateral	0.56 (0.23–1.34)	0.195		
Maximum tumor diameter (CM) (%)	< 5	Reference	0.190		
	5–10	1.99 (1.64–2.52)	0.969		
	≥10	1.45 (1.19–2.09)	0.079		
Number of tumors (%)	Single	Reference	0.775		
	Multiple	1.92 (1.52–2.64)			
Perioperative information					
Operative approach (%)	Open	Reference	0.019	Reference	0.025
	Laparoscopic	0.62 (0.28–0.74)		0.83 (0.48–0.91)	
Surgical duration (%)	< 180	Reference	< 0.001	Reference	< 0.001
	≥180	3.05 (2.02–4.62)		7.44 (4.02–13.78)	
Intraoperative blood loss (%)	< 400	Reference	< 0.001	
	≥400	2.78 (1.84–4.20)		

ECOG, eastern cooperative oncology group; BCLC, barcelona clinic liver cancer; SII, systemic immune-inflammation index; CONUT, controlling nutritional status; AFP, alpha-fetoprotein; EPI, early postoperative infection; OR, odds ratio. All ORs presented in this table were adjusted for study site in the multivariable logistic regression model.

Specifically, compared with patients with an ECOG performance status of 0–1, those with ECOG scores of 2–4 had a significantly increased risk of EPI (OR = 4.96, 95% CI: 2.73–9.00, *P* < 0.001). Patients with SII ≥600 had a higher risk of EPI than those with SII < 600 (OR = 2.73, 95% CI: 1.43–5.22, *P* = 0.023). Similarly, a CONUT score ≥3 was associated with a markedly increased risk of EPI (OR = 7.84, 95% CI: 3.46–15.34, *P* < 0.001).

Furthermore, compared with open surgery, laparoscopic surgery was associated with a lower risk of EPI (OR = 0.83, 95% CI: 0.48–0.91, *P* = 0.025). Longer operative duration (≥180 min) was significantly associated with an increased risk of EPI, with patients undergoing prolonged surgery having approximately a 7.44-fold higher risk compared with those with operative duration < 180 min (OR = 7.44, 95% CI: 4.02–13.78, *P* < 0.001).

### Development and validation of the INSEP model

Based on the independent risk factors identified above, an individualized risk prediction model was developed to accurately estimate the probability of EPI occurrence in elderly patients with HCC (Figure 4). To facilitate the integration of multiple predictors and provide individualized risk assessment, an INSEP nomogram was subsequently constructed.

**Figure 4:**
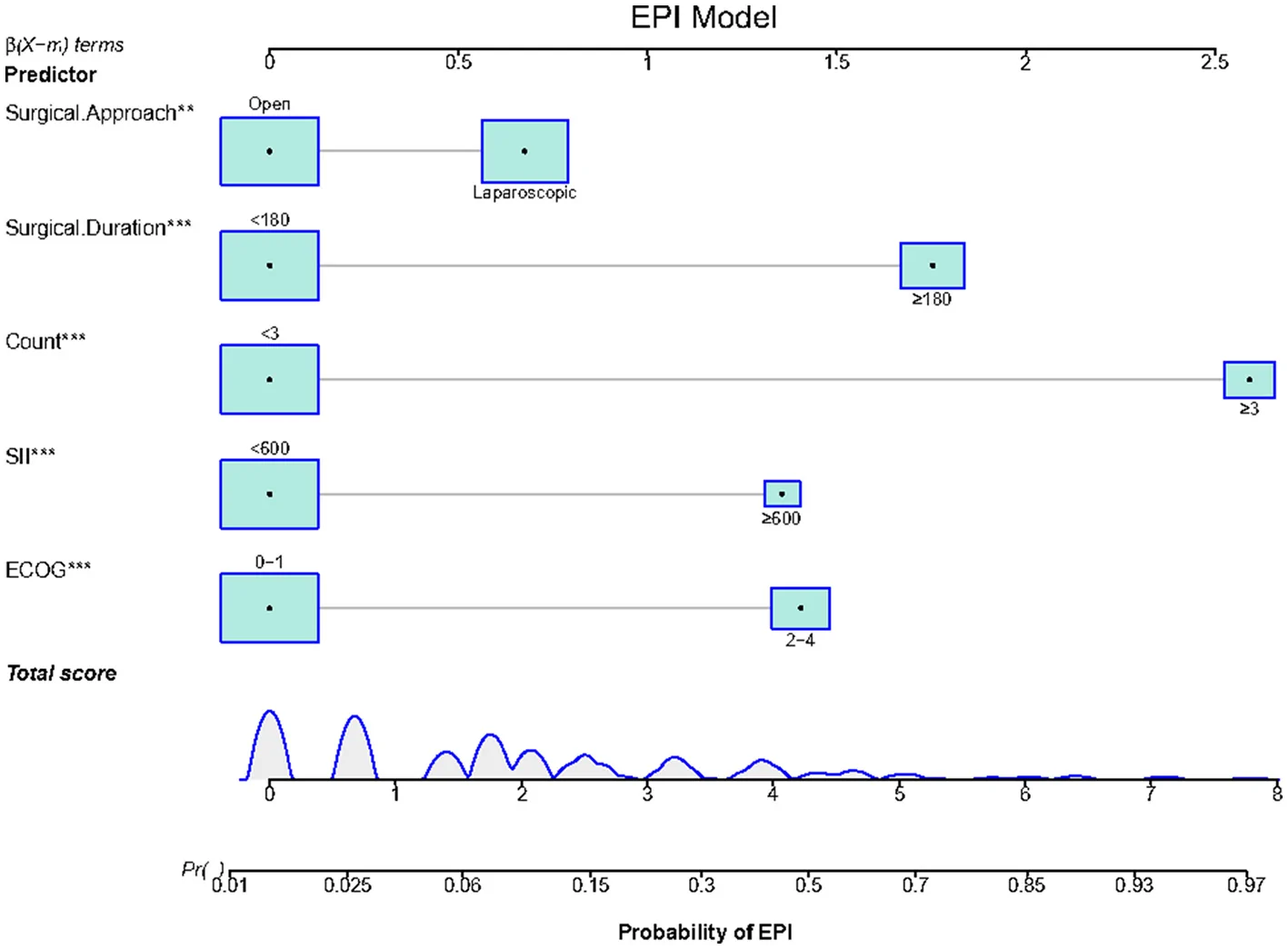
INSEP model predicting the occurrence of EPI in patients with elderly HCC patients; EPI, early postoperative infection.

The nomogram demonstrated excellent discriminatory performance, with AUC values of 0.870, 0.803, and 0.798 in the training dataset, internal validation dataset, and external validation dataset, respectively (Figures 5A–C). DeLong's test showed that the AUC values of the nomogram were significantly higher than those of each individual predictor in all datasets (all *P* < 0.05), indicating superior discrimination of the combined prediction model compared with any single predictor alone (Figures 5D–F).

**Figure 5:**
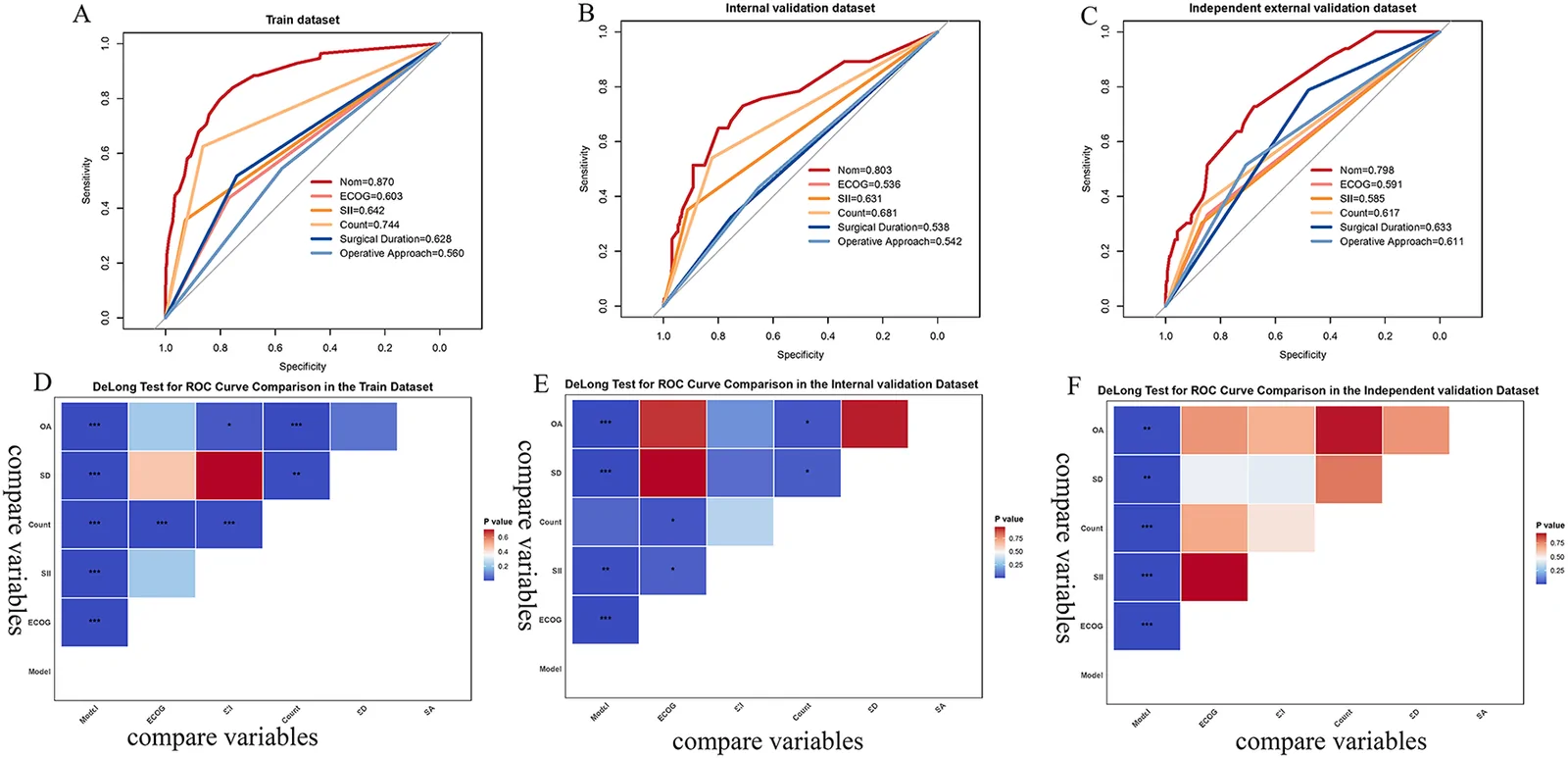
ROC curves of the INSEP model in the training dataset **(A)**, internal validation dataset **(B)**, and external validation dataset **(C)**; DeLong test comparisons of the INSEP model versus other predictive models in the training dataset **(D)**, internal validation dataset **(E)**, and external validation dataset **(F)**. ROC, receiver operating characteristic; AUC, area under the curve. ^*^represents *p* < 0.05, ^**^represents *p* < 0.01, ^***^represents *p* < 0.001. DeLong test was used to compare AUCs between models.

Calibration curves demonstrated good agreement between the predicted probabilities and the observed outcomes, with minimal overall deviation, indicating satisfactory calibration and reliability of the model. Specifically, points located above the diagonal reference line indicate that the model overestimated the risk, whereas points below the line indicate risk underestimation. In all validation datasets, the calibration curves closely approximated the ideal diagonal line, further supporting the robustness and potential clinical applicability of the INSEP nomogram (Figures 6A–C).

**Figure 6:**
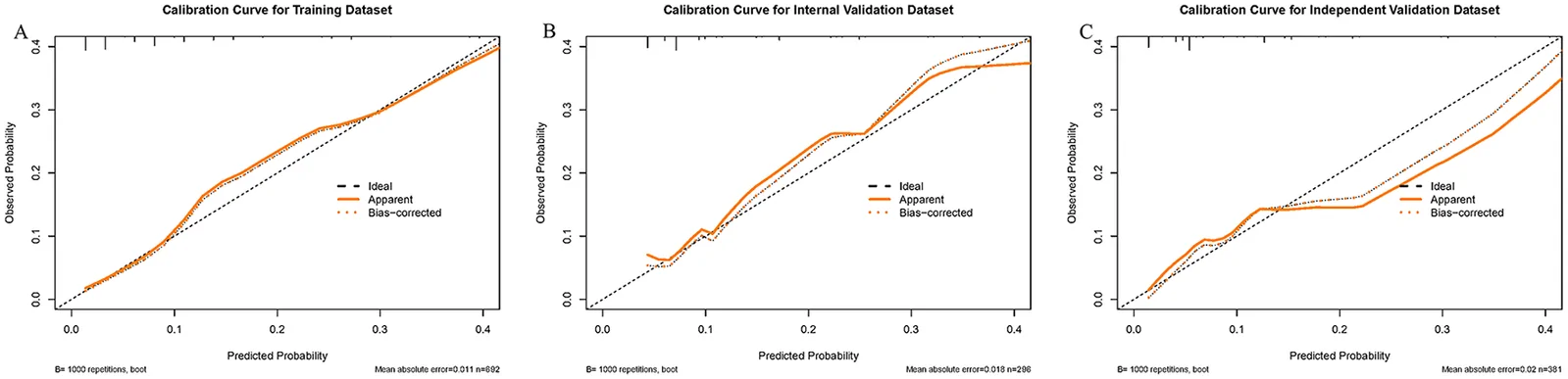
Calibration curves of the INSEP model for predicting EPI in the training dataset **(A)**, internal validation dataset **(B)**, and external validation dataset **(C)**; EPI, early postoperative infection.

### Clinical utility and net benefit analysis

DCA and CIC were performed to evaluate the clinical utility of the INSEP prediction model and quantify its potential net benefit. As shown in Figures 7A–C, within a clinically relevant range of threshold probabilities, the INSEP model achieved greater net benefit compared with the “treat-all” strategy, the “treat-none” strategy, and individual predictors.

**Figure 7:**
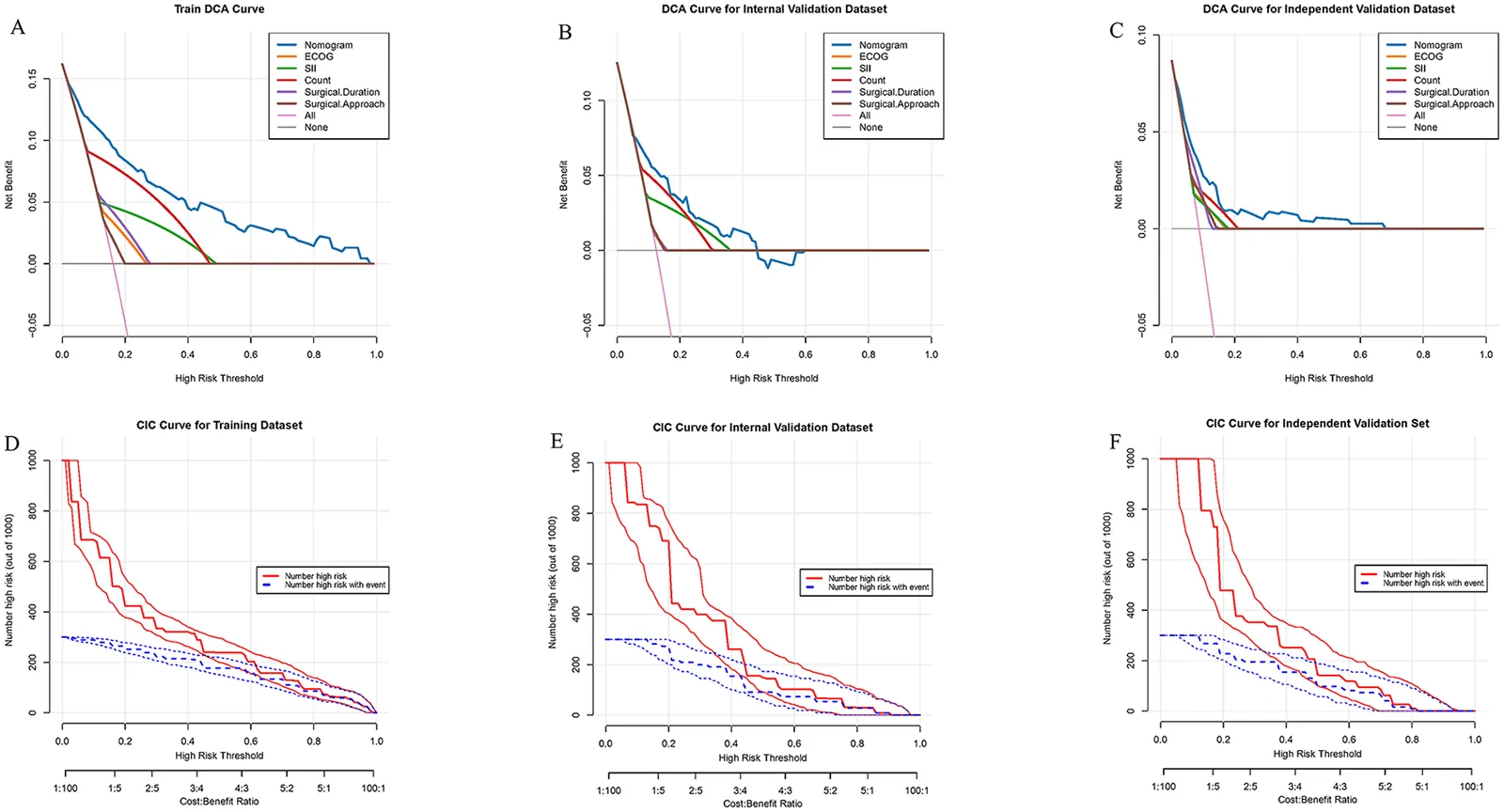
DCA and CIC of the INSEP model for predicting EPI. The net clinical benefit of the model was evaluated across different threshold probabilities. **(A–C)** Show the DCA results for the training dataset, internal validation dataset, and external validation dataset, respectively, while **(D–F)** present the corresponding CICs. DCA, Decision curve analysis; CIC, clinical impact curves; EPI, early postoperative infection.

The clinical impact curves further demonstrated that the INSEP model maintained a favorable ability to identify patients at high risk of EPI across a wide range of risk thresholds (Figures 7D–F). These findings suggest that the INSEP model provides substantial advantages in supporting clinical decision-making and improving the accuracy of individualized risk stratification.

Furthermore, these results indicate that a prediction model integrating multiple prognostic factors provides superior predictive performance compared with single-variable approaches and may serve as a practical decision-support tool for assisting clinicians in identifying elderly HCC patients who are at increased risk of EPI.

### Deployment of a web-based clinical decision support system

To facilitate the clinical implementation of the INSEP prediction model, we further deployed the model as an online web-based risk calculator, enabling clinicians to rapidly estimate the risk of EPI in elderly HCC patients based on individual clinical characteristics and laboratory parameters. The online calculator incorporates five independent risk factors, including surgical approach, operative duration, CONUT score, SII, and ECOG performance status (Figure 8). This web-based risk calculator is publicly accessible at: https://shao1234567.shinyapps.io/epi-risk-predictor/.

**Figure 8:**
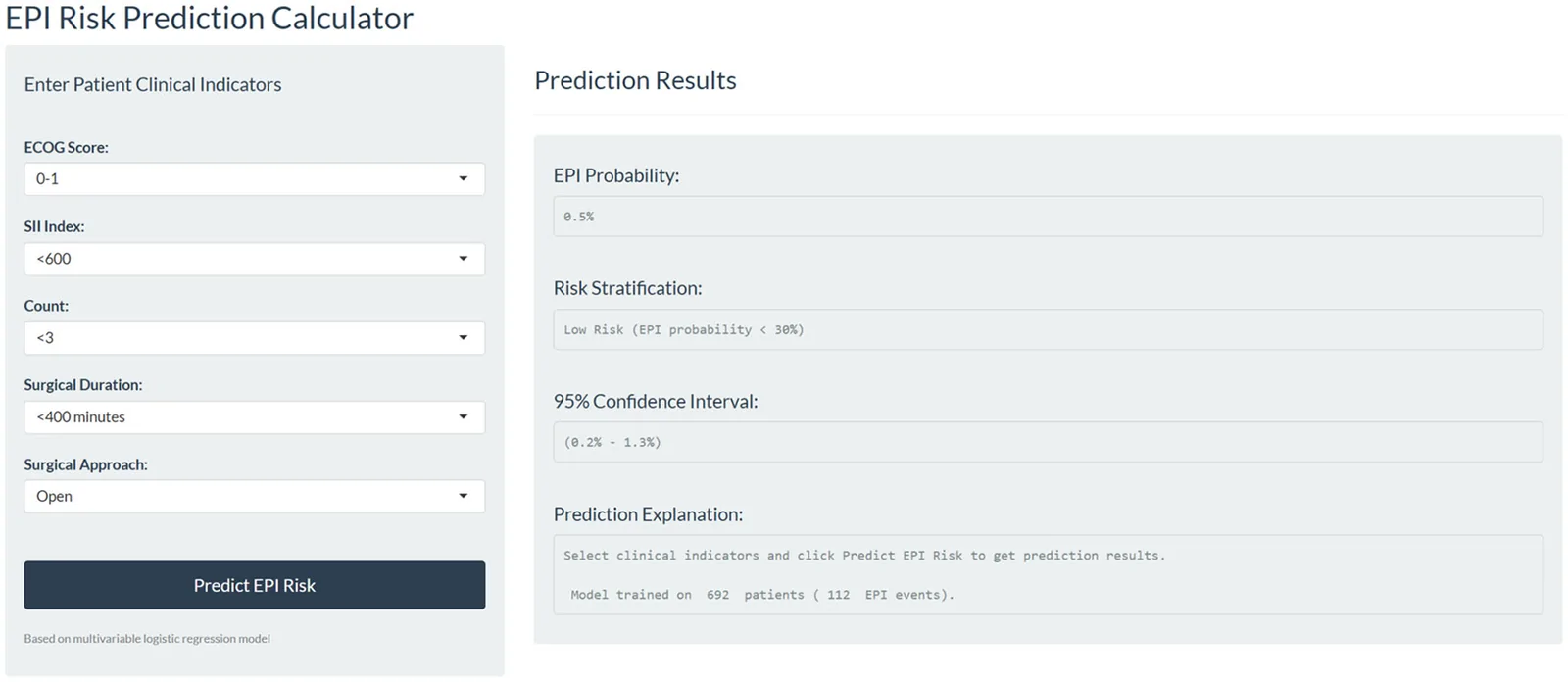
Deployment of a web-based clinical decision support system based on the INSEP model.

### Clinical scenario demonstration and user experience

To intuitively demonstrate the practical value of the INSEP online decision-support system for EPI risk assessment, two representative cases from the external validation dataset were selected for illustration (Supplementary Table 6).

In the low-risk case, the model predicted an EPI probability of 3.3%, and the patient did not develop any postoperative infectious events. In contrast, in the high-risk case, the predicted infection probability was 97.0%, and the patient developed pneumonia on postoperative day 5, which was consistent with the model prediction. Screenshots of the online calculator interface and corresponding risk prediction results are presented in Supplementary Figure 5.

Furthermore, three independent hepatobiliary surgeons, with 6, 8, and 10 years of clinical experience, respectively, were invited to evaluate the usability of the INSEP online calculator. All three physicians considered the tool easy to operate, with an estimated assessment time of approximately 25–60 s per patient. They reported that the interface was intuitive and that the risk prediction outputs were clear and easy to interpret. The physicians indicated that the risk stratification results could facilitate preoperative patient–physician communication and support perioperative clinical decision-making. No system errors or usability barriers were identified during the testing process. One physician suggested that incorporating specific infection types (e.g., pneumonia and surgical site infection) into the model output might further enhance its clinical utility (Supplementary Table 7). Collectively, these findings support the INSEP online calculator as a practical individualized risk stratification tool for assessing the risk of early postoperative infection among elderly HCC patients undergoing curative hepatectomy.

## Discussion

In this study, we developed and externally validated an inflammation–nutrition–surgery-based predictive model for EPI in elderly HCC patients undergoing curative hepatectomy. Using a large multicenter dataset and a robust variable selection strategy based on LASSO and multivariable logistic regression analyses, we identified several independent predictors of EPI, including ECOG ≥2, SII ≥600, CONUT score ≥3, laparoscopic vs. open surgical approach, and surgical duration ≥180 min. Patients with these high-risk features exhibited a significantly higher incidence of postoperative EPI, which was further associated with adverse clinical outcomes, including prolonged hospital stay, increased ICU admission rates, and greater severity of postoperative complications.

Our findings support and extend previous evidence demonstrating a close association between poor preoperative nutritional status, a heightened systemic inflammatory state, and an increased risk of adverse postoperative outcomes in patients with malignancies (24–27). A recent multicenter retrospective study involving 210 patients with gallbladder cancer undergoing curative resection reported that an elevated systemic SII was independently associated with worse short-term outcomes and poorer long-term prognosis (28). Moreover, a combined model incorporating SII and hemoglobin achieved better predictive performance than either marker alone, highlighting the synergistic prognostic value of inflammatory and nutritional status (24, 29). Consistent with these findings, our study demonstrated that elevated preoperative SII and a higher CONUT score were independently associated with an increased risk of EPI in elderly HCC patients, supporting the importance of integrating inflammatory and nutritional parameters for perioperative risk assessment.

Mechanistically, an elevated SII reflects enhanced neutrophil- and platelet-mediated inflammatory activity together with impaired lymphocyte-mediated immune function. Excessive activation of the innate immune response may promote tissue injury and immune dysregulation, whereas lymphopenia compromises host antimicrobial defense, thereby increasing susceptibility to postoperative infections (25). Meanwhile, poor nutritional status, as reflected by a high CONUT score, may further aggravate immunosuppression by impairing immune cell proliferation, protein synthesis, and tissue repair. Consequently, patients with concurrent systemic inflammation and malnutrition may develop a state characterized by “high inflammation, impaired immunity, and poor nutritional reserve,” which substantially increases the risk of postoperative infectious complications following major surgery (26, 27, 30).

Notably, these associations may be particularly pronounced in elderly patients because aging is accompanied by two hallmark biological processes, namely immunosenescence and inflammaging (31–33). With advancing age, immune function progressively declines, while chronic low-grade inflammation persists, rendering elderly individuals more susceptible to exaggerated inflammatory responses and immune dysregulation following surgical stress (34). In addition, elderly patients often have reduced physiological reserve and a greater burden of comorbidities, further limiting their ability to recover from surgical trauma and increasing their vulnerability to postoperative infectious complications (28). Therefore, inflammatory and nutritional biomarkers may capture not only conventional inflammatory and nutritional status but also age-related physiological vulnerability in elderly HCC patients.

In addition, this study also found that a higher ECOG performance status score (2–4) was significantly associated with the occurrence of EPI. As an important indicator reflecting overall functional status and physiological reserve, the ECOG score has been widely used in prognostic assessment of cancer patients (35–37). A higher ECOG score generally indicates limited physical activity, poorer baseline condition, and reduced tolerance to surgical stress (38, 39).

Furthermore, patients with poor performance status are often accompanied by impaired immune function and malnutrition. Impaired immune function weakens the host's ability to eliminate pathogens, while malnutrition may compromise immune cell proliferation and functional recovery, thereby increasing the risk of postoperative infection. In addition, reduced perioperative mobility in patients with higher ECOG scores may further contribute to an increased incidence of pulmonary infections and other complications, which is consistent with the findings observed in this study (40, 41). Therefore, the ECOG score not only serves as a simple tool for assessing baseline patient status, but may also be combined with inflammatory and nutritional indicators to enable early identification of patients at high risk of postoperative infection.

In this study, we found that laparoscopic surgery was associated with a lower risk of EPI compared with open surgery, while prolonged surgical duration (>3 h) was significantly associated with an increased risk of EPI in elderly patients with HCC. These findings highlight the important role of operative approach and surgical burden in the development of perioperative infections. Minimally invasive approaches, such as laparoscopic surgery, are generally associated with reduced surgical trauma, shorter tissue exposure time, and a more controlled intraoperative environment. These advantages may lead to a less pronounced systemic inflammatory response and better preservation of postoperative immune function (42, 43). In addition, laparoscopic procedures are typically associated with less intraoperative blood loss and less extensive tissue manipulation, which may further reduce the risk of postoperative infectious complications (44–46). In contrast, open surgery often involves greater surgical trauma, prolonged exposure of tissues, and more complex operative procedures, all of which may contribute to increased susceptibility to postoperative infection.

On the other hand, prolonged surgical duration is often indicative of increased procedural complexity and a higher perioperative stress burden. Extended operative time may increase the risk of intraoperative contamination and sustain systemic inflammatory activation, thereby exacerbating postoperative immunosuppression (47–49). Furthermore, prolonged anesthesia exposure and ischemia–reperfusion injury may impair immune recovery and delay postoperative healing (50, 51). These effects may be particularly pronounced in elderly patients, who often exhibit reduced physiological reserve and immunosenescence. Taken together, our findings suggest that optimizing surgical strategies—such as prioritizing minimally invasive approaches when feasible and improving operative efficiency to reduce surgical duration—may represent important avenues for reducing the risk of postoperative infection in elderly HCC patients.

Furthermore, the web-based INSEP calculator demonstrated good clinical applicability in representative clinical scenarios. By providing rapid and individualized risk estimation, the tool may facilitate preoperative risk stratification, perioperative management, and clinical decision-making for elderly HCC patients. Compared with conventional prediction models, the INSEP calculator combines favorable predictive performance with ease of use, supporting its potential for routine clinical implementation. Nevertheless, further prospective multicenter studies are warranted to evaluate its real-world effectiveness and clinical impact.

Several limitations of this study should be acknowledged. First, this was a retrospective study. Although a relatively large sample size and multicenter validation were employed, selection bias and information bias could not be completely eliminated. Prospective studies are therefore required to further validate the reliability of the model. Second, the study population was derived predominantly from Chinese patients. Although the INSEP model demonstrated good discriminative ability in the independent external validation dataset (AUC = 0.798), its applicability to other ethnic populations and healthcare systems requires further validation using larger international multicenter datasets. Third, the model was primarily developed using preoperative inflammatory and nutritional indicators together with perioperative variables. More detailed intraoperative parameters, such as real-time physiological changes and anesthesia-related factors, as well as dynamic postoperative indicators, were not included. The absence of these variables may limit the model's ability to fully characterize the mechanisms and dynamic evolution of EPI. Future studies integrating multidimensional clinical information may further improve prediction accuracy. Fourth, several continuous variables were converted into categorical variables according to optimal cutoff values derived from ROC curve analysis to improve model interpretability and facilitate clinical application. Although restricted cubic spline analysis demonstrated no significant nonlinear association between SII or operative duration and EPI risk, categorization of continuous variables inevitably results in some loss of information. In addition, the ROC-derived optimal cutoff values for SII (597) and operative duration (178 min) were rounded to 600 and 180 min, respectively, to enhance clinical practicality. The stability of these cutoff values should be further confirmed in larger prospective datasets. Fifth, baseline characteristics differed between the model development dataset and the external validation dataset, reflecting the inherent heterogeneity in patient populations, perioperative management strategies, and surgical practices across different medical centers. To minimize the potential influence of center effects, study center was included as an adjustment variable in the multivariable logistic regression analysis. The results showed that study center was not independently associated with EPI and that adjustment for center effects resulted in only minimal changes in the estimated effects of the predictors. Moreover, despite the observed inter-center heterogeneity, the INSEP model maintained stable predictive performance in the external validation dataset, suggesting satisfactory generalizability beyond specific institutional settings. Sixth, large-scale prospective evidence evaluating the real-world implementation of the INSEP model is still lacking. Future multicenter prospective studies are warranted to further assess its robustness, generalizability, and clinical impact on perioperative management and patient outcomes across diverse clinical settings. Finally, although independent hepatobiliary surgeons provided favorable feedback regarding the usability and clinical practicality of the INSEP online calculator, one physician suggested that the current model predicts only the overall risk of EPI without distinguishing specific infection types. Future studies incorporating more detailed infection phenotypes and developing multitask prediction models may further enhance its value for infection-specific risk prediction and clinical decision support.

## 5 Conclusion

This study developed and validated a comprehensive predictive model (INSEP) based on inflammatory, nutritional, and surgical factors for estimating the risk of early postoperative infection (EPI) in elderly patients undergoing curative hepatectomy for hepatocellular carcinoma (HCC). The model demonstrated stable and excellent discriminative ability and calibration performance across multicenter datasets, outperforming conventional single biomarkers. The INSEP model provides a simple and reliable tool for preoperative risk stratification, enabling clinicians to identify high-risk patients and implement individualized perioperative management strategies, thereby potentially improving patient outcomes. Future prospective studies are still required to further validate its clinical utility.

## Data Availability

The raw data supporting the conclusions of this article will be made available by the authors, without undue reservation.

## Supplementary material

Supplementary Figure 1 Restricted cubic spline analysis of the associations between SII index, surgical duration, and EPI. RCS, restricted cubic spline; SII, systemic immune-inflammation index; EPI, early postoperative infection; OR, odds ratio; CI, confidence interval.

Supplementary Figure 2 ROC curve analysis of SII index and surgical duration for predicting EPI. ROC, Receiver operating characteristic; SII, systemic immune-inflammation index; EPI early postoperative complications.

Supplementary Figure 3 Common types of EPI **(A)** and their associated pathogens **(B)**; EPI, early postoperative infections.

Supplementary Figure 4 Assessment of multicollinearity among candidate variables using variance inflation factor (VIF).

Supplementary Figure 5 Demonstration of the INSEP online calculator input and output interfaces for a low-riskcase **(Left)** and a high-risk case **(Right)**.

Supplementary Table 1 Definitions of postoperative infections.

Supplementary Table 2 Postoperative clinical outcomes stratified by EPI.

Supplementary Table 3 Candidate variables included in LASSO regression analysis.

Supplementary Table 4 Variables retained by LASSO regression at λ.min.

Supplementary Table 5 Comparison of odds ratios for EPI predictors before and after adjustment for center effects.

Supplementary Table 6 Clinical characteristics and outcomes of two representative cases from the external validation dataset.

Supplementary Table 7 Usability feedback from three independent hepatobiliary surgeons on the INSEP online calculator.
