# Supplementary material for: An inflammation-nutrition-surgery-based INSEP model and a web-based clinical decision support system for predicting early postoperative infection in elderly patients with hepatocellular carcinoma: a multicenter retrospective study
Source: Front Nutr. 2026 Sep 22;13:1883480. doi: 10.3389/fnut.2026.1883480 (PMC13638509; doi:10.3389/fnut.2026.1883480)
Supplement: Supplementary file 1 [file Supplementary_file_1.DOCX]

**Supplementary table 1. Definitions of postoperative infections.**

| **Infection type** | Diagnostic criteria |
| --- | --- |
| Surgical site infection (SSI) | The diagnosis of SSI was based on the Centers for Disease Control and Prevention/National Healthcare Safety Network (CDC/NHSN) criteria for surgical site infections. SSI was defined as an infection occurring within 7 days after surgery and meeting at least one of the following criteria: (1) purulent drainage from the incision site, surgical area, or organ/space drain; (2) isolation of pathogenic microorganisms from aseptically obtained specimens collected from the incision, deep tissues, or involved organs/spaces; (3) clinical signs suggestive of infection, including incision pain, tenderness, localized erythema, swelling, fever, or wound dehiscence, with infection confirmed by the attending physician; or (4) evidence of infection involving the surgical site, related organs, or spaces confirmed by imaging examinations or direct examination. According to the depth and extent of involvement, SSI was further classified into superficial incisional SSI, deep incisional SSI, and organ/space SSI. |
| Postoperative pneumonia (PP) | The diagnosis of postoperative pneumonia was based on the CDC/NHSN pneumonia criteria. PP was defined as pneumonia occurring within 7 days after surgery and requiring both: (1) new or progressive infiltrates, consolidation, cavitation, or other infectious changes on chest imaging; and (2) at least one clinical or laboratory indicator of infection, including fever, abnormal white blood cell count (leukocytosis or leukopenia), purulent respiratory secretions, or worsening respiratory symptoms. Microbiological results from respiratory specimens, including sputum, tracheal aspirates, or bronchoalveolar lavage fluid, were considered when available to support the diagnosis. |
| Urinary tract infection (UTI) | The diagnosis of UTI was based on the CDC/NHSN urinary tract infection criteria. UTI was defined as an infection occurring within 7 days after surgery, accompanied by urinary tract-related symptoms or systemic manifestations of infection, including fever, dysuria, urinary frequency, urgency, suprapubic pain, or costovertebral angle tenderness. Urine cultures were collected using standardized procedures, and the diagnosis was determined according to microbiological findings combined with clinical manifestations. |
| Intra-abdominal infection (IAI) | IAI was diagnosed based on a combination of clinical manifestations, laboratory findings, imaging examinations, and microbiological results. IAI was defined as an infection occurring within 7 days after surgery and meeting at least one of the following criteria: (1) persistent fever, abdominal pain, peritoneal irritation signs, or other clinical manifestations suggestive of intra-abdominal infection; (2) elevated inflammatory markers, such as increased white blood cell count or C-reactive protein levels; (3) imaging evidence of infectious changes, including intra-abdominal abscess, infected fluid collection, or other abnormal fluid accumulation; or (4) isolation of pathogenic microorganisms from abdominal drainage fluid, puncture specimens, or surgical specimens. |
| Sepsis | Postoperative sepsis was defined as sepsis occurring within 7 days after surgery. The diagnosis required suspected or confirmed infection accompanied by acute organ dysfunction, defined as an increase in SOFA score ≥2 points according to the Sepsis-3 criteria. The underlying infection source was determined according to CDC/NHSN criteria whenever applicable. |

**Note**: All of the above postoperative infection types were defined as newly developed infectious events occurring within 7 days after hepatectomy. All infection cases were diagnosed according to standardized criteria across participating centers. The diagnosis of infection was comprehensively determined based on clinical manifestations, laboratory findings, microbiological results, and imaging examinations. The specific diagnostic criteria were based on the principles of hospital infection surveillance definitions established by the Centers for Disease Control and Prevention (CDC)/National Healthcare Safety Network (NHSN). For other less common infection types, diagnoses were independently confirmed by at least two experienced clinicians at each participating center according to internationally accepted diagnostic criteria, ensuring consistency in infection classification among different centers. Abbreviations: EPI, early postoperative infection; SSI, surgical site infection; PP, postoperative pneumonia; UTI, urinary tract infection; IAI, intra-abdominal infection.

**Supplementary table 2. Postoperative clinical outcomes stratified by EPI.**

| **Variables** |  | Total  (N = 1369) | Non-EPI  (N = 1187) | EPI  (N = 182) | *P*  Value |
| --- | --- | --- | --- | --- | --- |
| Length of Hospital Stay (mean (SD)) |  | 12.023 (7.596) | 11.107 (5.359) | 17.995 (14.370) | <0.001 |
| ICU admission postoperatively (%) | No | 1261 (92.11) | 1134 (95.53) | 127 (69.78) | <0.001 |
|  | Yes | 108 (7.89) | 53 (4.47) | 55 (30.22) |  |
| Minor complications (%) | No | 949 (69.32) | 851 (71.69) | 98 (53.85) | <0.001 |
|  | Yes | 420 (30.68) | 336 (28.31) | 84 (46.15) |  |
| Major complications (%) | No | 1089 (79.55) | 997 (83.99) | 92 (50.55) | <0.001 |
|  | Yes | 280 (20.45) | 190 (16.01) | 90 (49.45) |  |

**Abbreviations:** ICU, Intensive Care Unit Admission; Minor complications, Clavien-Dindo Grade I-II; Major complications, Clavien-Dindo Grade III; EPI, Early Postoperative Infection.

**Supplementary table 3. Candidate variables included in LASSO regression analysis.**

| **Classification** | **Candidate variable** |
| --- | --- |
| Demographic | Age、Sex、BMI |
| Clinical characteristics | ECOG、Smoke、Drink、DM、History of liver disease、Cirrhosis、Child-Pugh grade、BCLC stage |
| Laboratory variables | SII、CONUT、Preoperative AFP |
| Tumor characteristics | Tumor location、Maximum tumor diameter、Number of tumors |
| Surgical variables | Operative Approach、Surgical duration、Intraoperative blood loss、Intraoperative blood transfusion |

**Note:** All variables listed in this table were entered into the LASSO regression analysis as candidate predictors for variable selection.

**Abbreviations:** BMI, body mass index; ECOG, Eastern Cooperative Oncology Group Performance Status; DM, diabetes mellitus; BCLC, Barcelona Clinic Liver Cancer; SII, systemic immune-inflammation index; CONUT, controlling nutritional status; AFP, alpha-fetoprotein.

**Supplementary table 4. Variables retained by LASSO regression at λ.min.**

| Variable | LASSO coefficient (λ.min = 0.00682) |
| --- | --- |
| ECOG | 0.1343 |
| Drinking history | 0.0274 |
| Child-Pugh grade | 0.0385 |
| BCLC stage | 0.0122 |
| SII | 0.1579 |
| CONUT | 0.3347 |
| Preoperative AFP | 0.1208 |
| Tumor location | -0.0026 |
| Maximum tumor diameter | 0.0164 |
| Number of tumors | -0.0075 |
| Operative Approach | 0.0535 |
| Surgical duration | 0.1482 |
| Intraoperative blood loss | 0.0149 |

**Note:** The optimal penalty parameter was selected using the λ.min criterion based on 10-fold cross-validation (λ.min = 0.00682). The corresponding λ.1se was 0.04385. The coefficients presented are those of variables retained at λ.min.

**Abbreviations:** ECOG, Eastern Cooperative Oncology Group Performance Status; BCLC, Barcelona Clinic Liver Cancer; SII, systemic immune-inflammation index; CONUT, controlling nutritional status; AFP, alpha-fetoprotein.

**Supplementary table 5. Comparison of odds ratios for EPI predictors before and after adjustment for center effects.**

| **Variables** | **OR Comparison** | **OR before adjustment** | **OR after adjustment** | **Relative change (%)** | **95% CI (adjusted)** | **P value (adjusted)** |
| --- | --- | --- | --- | --- | --- | --- |
| **Demographic data** |  |  |  |  |  |  |
| ECOG (%) | 0-1 | Reference | Reference | — | Reference | ＜0.001 |
|  | 2-4 | 5.13 | 4.96 | 3.3 | 4.96（2.73-9.00） |  |
| Drink (%) | No | Reference | Reference | — |  |  |
|  | Yes | 1.21 | 1.18 | 2.5 |  |  |
| Child Pugh Grade (%) | A | Reference | Reference | — |  |  |
|  | B | 1.85 | 1.82 | 1.6 |  |  |
| BCLC Stage (%) | 0 | Reference | Reference | — |  |  |
|  | A | 1.78 | 1.75 | 1.7 |  |  |
|  | B | 2.39 | 2.35 | 1.7 |  |  |
| **Laboratory Data** |  |  |  |  |  |  |
| SII (%) | <600 | Reference | Reference | — | Reference | 0.023 |
|  | ≥600 | 2.75 | 2.73 | <1 | 2.73(1.43-5.22) |  |
| CONUT (%) | <3 | Reference | Reference | — | Reference | ＜0.001 |
|  | ≥3 | 7.88 | 7.84 | <1 | 7.84(3.46-15.34) |  |
| Preoperative AFP (%) | <400 | Reference | Reference | — |  |  |
|  | ≥ 400 | 2.89 | 2.85 | 1.4 |  |  |
| **Imaging Data** |  |  |  |  |  |  |
| Tumor Location (%) | right lobe | Reference | Reference | — |  |  |
|  | left lobe | 1.64 | 1.62 | 1.2 |  |  |
|  | bilateral | 0.56 | 0.55 | 1.8 |  |  |
| Maximum Tumor Diameter (CM) (%) | <5 | Reference | Reference | — |  |  |
|  | 5-10 | 1.99 | 1.96 | 1.5 |  |  |
|  | ≥10 | 1.45 | 1.43 | 1.4 |  |  |
| Number of Tumors (%) | single | Reference | Reference | — |  |  |
|  | multiple | 1.92 | 1.89 | 1.6 |  |  |
| **Perioperative information** |  |  |  |  |  |  |
| Operative Approach (%) | open | Reference | Reference | — | Reference | 0.025 |
|  | laparoscopic | 0.83 | 0.83 | <1 | 0.83(0.48-0.91) |  |
| Surgical Duration (%) | <180 | Reference | Reference | — | Reference | <0.001 |
|  | ≥180 | 7.47 | 7.44 | <1 | 7.44(4.02-13.78) |  |
| Intraoperative Blood Loss (%) | <400 | Reference | Reference | — |  |  |
|  | ≥400 | 2.78 | 2.74 | 1.4 |  |  |
| **Study center** | A | Reference | Reference |  |  |  |
|  | B | 1.12 | — | — | 1.12 (0.69–1.82) | 0.642 |

**Abbreviations:** Note: OR, odds ratio; CI, confidence interval; ECOG, Eastern Cooperative Oncology Group performance status; SII, systemic immune-inflammation index; CONUT, controlling nutritional status; AFP, alpha-fetoprotein; BCLC, Barcelona Clinic Liver Cancer staging system. ORs before adjustment were derived from the multivariable logistic regression model without inclusion of the study site covariate. ORs after adjustment were derived from the multivariable logistic regression model with study site included as a covariate. Relative change (%) was calculated as |OR after adjustment − OR before adjustment| / OR before adjustment × 100%. **Note:** Study Site A: The First Hospital of Jilin University; Study Site B: The First Affiliated Hospital of Zhengzhou University.

**Supplementary Table 6**. **Clinical characteristics and outcomes of two representative cases from the external validation dataset.**

| **Assessment Parameter** | **Case A (Low Risk)** | **Case B (High Risk)** |
| --- | --- | --- |
| Patient Baseline | Elderly HCC patient | Elderly HCC patient |
| Operation Approach | Laparoscopic | Open |
| Surgical Duration | 120 minutes | 210 minutes |
| CONUT Score | 1 (Well-nourished) | 4 (Malnourished) |
| SII | 350 (Low inflammation) | 850 (High inflammation) |
| ECOG PS Score | 0 | 2 |
| INSEP‑predicted EPI risk | 3.3% (Low risk) | 97.0% (High risk) |
| Postoperative infectious complication | None | Pneumonia |
| Length of Stay | 7 | 21 |
| ICU admission | No | Yes |
| Postoperative recovery | Uneventful | Complicated |

**Abbreviations:** HCC, hepatocellular carcinoma; CONUT, Controlling Nutritional Status; SII, systemic immune‑inflammation index; ECOG PS, Eastern Cooperative Oncology Group performance status; EPI, early postoperative infection; ICU, intensive care unit.

**Supplementary Table 7. Usability feedback from three independent hepatobiliary surgeons on the INSEP online calculator.**

| **Assessment Parameter** | **Surgeon A (8 years)** | **Surgeon B (10 years)** | **Surgeon C (6 years)** |
| --- | --- | --- | --- |
| Operation time per case | 30–45 seconds | 40–60 seconds | 25–40 seconds |
| Interface design | Intuitive and concise | Generally intuitive, minor learning curve | Very intuitive, no learning curve needed |
| Risk output readability | Very easy to interpret | Easy to interpret with basic explanation | Extremely easy to interpret |
| Usefulness for preoperative communication | Very helpful | Helpful | Very helpful |
| Usefulness for perioperative decision‑making | Very helpful | Helpful, but suggests adding infection type | Very helpful |
| System errors | None identified | None identified | None identified |
| Usability barriers | None identified | None identified | None identified |
| Overall recommendation | Strongly recommend | Recommend with minor suggestions | Strongly recommend |
